# New Curriculum Guidelines for Undergraduate Nursing Education: a historic achievement for Brazilian nursing

**DOI:** 10.1590/0034-7167.20267907

**Published:** 2026-10-02

**Authors:** Célia Alves Rozendo, Jacinta de Fatima Sena da Silva, Kênia Lara da Silva

**Affiliations:** I Universidade Federal de Alagoas. National Director of Education, Brazilian Nursing Association (2025-2028 Administration). Alagoas, Maceió, Brazil; II Fiocruz Brasília, Faculty Member, Master’s Program in Public Health Policies, Angicos, and PSAT. Universidade de Brasília, Department of Collective Health. Researcher and Collaborating Faculty Member. National President of the Brazilian Nursing Association (2025-2028 Administration). Brasília, Federal District, Brazil; III Universidade Federal de Minas Gerais, School of Nursing. National Director of Nursing Studies and Research, Brazilian Nursing Association (2025-2028 Administration). Belo Horizonte, Minas Gerais, Brazil

In 2012, Belém, Pará, hosted the 13^th^ National Seminar on Guidelines for Nursing Education (13^th^ SENADEn - *Seminário Nacional de Diretrizes para a Educação em Enfermagem*), which marked the beginning of discussions on the revision of the Brazilian National Curriculum Guidelines for Undergraduate Nursing Programs (DCN/ENF - *Diretrizes Curriculares Nacionais para os Cursos de Graduação em Enfermagem*) under the central theme “A Decade of National Curriculum Guidelines: Achievements and Challenges”. A considerable period elapsed between the seminar and the publication of the new DCN/ENF, which occurred on May 19, 2026, through Resolution CES/CNE 1/2026^(1)^.

The result of broad participation and collective debate under the leadership of the Brazilian Nursing Association (ABEn - *Associação Brasileira de Enfermagem*), this milestone represents a fundamental achievement for nursing by establishing the foundations for excellence in nursing education. Every text is shaped by its context. Thus, the development of DCN/ENF, like any political process, mobilized multiple social actors, fostered extensive debate, and faced tensions, disputes, and challenges in defense of education and health as universal rights and inalienable public goods. Therefore, the new DCN/ENF are the product of a collective effort, reflecting the aspiration to transform nursing education and embodying the advances that were possible within a context of competing interests that was not always favorable but nevertheless stimulated the movements and advocacy efforts that culminated in this historic achievement.

The new DCN/ENF provide an opportunity to address the profound social, health, scientific, and educational transformations of contemporary society. Among their principal advances are the exclusive provision of undergraduate nursing education through face-to-face instruction, a milestone established by Decree 12,456 of May 19, 2025^(2)^, reflecting ABEn’s longstanding commitment to in-person nursing education as an ethical imperative for ensuring high-quality professional training; the recognition of the Unified Health System as the structural foundation of nursing education; the affirmation of nursing care as the core purpose of nurses’ professional practice, centered on addressing the population’s health needs and thereby strengthening the professional identity of nursing; and the incorporation of principles that respond to contemporary demands for education that is ethical, critical, politically engaged, socially accountable, and committed to democracy, citizenship, human dignity, and the reduction of social inequalities and exclusion.

Furthermore, the new DCN/ENF establish five areas of training and define the essential content required to preserve nurses’ technical and scientific autonomy, while explicitly emphasizing the need to ensure philosophical, theoretical, and methodological foundations in the development of each program’s Political-Pedagogical Project (PPP). They also reinforce the inseparability of teaching, research, and extension activities, as well as the integration of education, health services, and the community, together with the articulation between theory and practice.

Technical and scientific rigor is emphasized to promote the education of future nurses who are capable of responding to the population’s health needs and to the demands of the territories in which they practice, reaffirming nursing as a social practice. In this regard, the new DCN/ENF specify that the PPP must promote both theoretical teaching-learning activities and integrated theoretical-practical teaching-learning activities, highlighting the complementary nature of these educational components. It is important to note that theoretical-practical teaching-learning activities must begin in the first semester of the program and take place in diverse, real-world practice settings, accounting for at least 20% of the program’s total workload. Mandatory supervised clinical training, in turn, must represent 30% of the total program workload and be completed during the final two or three semesters.

In the year marking the centennial of ABEn, the publication of the new DCN/ENF represents a significant advance and a historic achievement for Brazilian nursing, reflecting the association’s longstanding commitment and leadership in matters related to nursing education and professional training. This achievement is the result of collective mobilization and sustained efforts in support of high-quality education, while also representing resistance to the commercialization of higher education. It reaffirms that victories are achieved only through collective struggle and that the future and strength of Brazilian nursing depend on continued mobilization in defense of life, health, and education.
