# Supplementary material for: New Curriculum Guidelines for Undergraduate Nursing Education: a historic achievement for Brazilian nursing
Source: Rev Bras Enferm. 2026 Oct 2;79:e7907. doi: 10.1590/0034-7167.20267907 (PMC13631969; doi:10.1590/0034-7167.20267907)
Supplement: Translation

# Translation: Novas Diretrizes Curriculares para a Graduação em Enfermagem: uma conquista histórica para a enfermagem brasileira

**DOI:** 10.1590/0034-7167.20267907pt

**Authors:** Célia Alves Rozendo, Jacinta de Fatima Sena da Silva, Kênia Lara da Silva

**Affiliations:** I Universidade Federal de Alagoas. Diretora Nacional de Educação da Associação Brasileira de Enfermagem (Gestão 2025-2028). Alagoas, Maceió, Brasil; II Fiocruz Brasília, Docente do Mestrado em Políticas Públicas de Saúde, Angicos e PSAT. Universidade de Brasília, Departamento de Saúde Coletiva. Pesquisadora e Docente Colaboradora. Presidenta Nacional da Associação Brasileira de Enfermagem (Gestão 2025-2028). Brasília, Distrito Federal, Brasil; III Universidade Federal de Minas Gerais, Escola de Enfermagem. Diretora Nacional de Estudos e Pesquisas em Enfermagem da Associação Brasileira de Enfermagem (Gestão 2025-2028). Belo Horizonte, Minas Gerais, Brasil

Em 2012, Belém do Pará foi o cenário do 13º Seminário Nacional de Diretrizes para a Educação em Enfermagem (13º SENADEn), que marcou o início das discussões sobre a revisão das Diretrizes Curriculares Nacionais para os Cursos de Graduação em Enfermagem (DCN/ENF), tendo como tema central “Uma Década de Diretrizes Curriculares Nacionais: conquistas e desafios”. Passou-se um longo tempo entre a realização deste SENADEn e a publicação das novas DCN/ENF, ocorrida no dia 19 de maio de 2026 por meio da Resolução CES/CNE n^o^ 1/2026^(1)^.

Resultado de ampla participação e debate coletivo sob a liderança da Associação Brasileira de Enfermagem (ABEn), este marco representa uma conquista fundamental para a enfermagem, apresentando as bases para uma formação de excelência. Todo texto traz em si um contexto. Assim, a construção das DCN/ENF, como todo processo político, mobilizou diversos atores sociais, produziu debates e enfrentou tensionamentos, disputas e desafios em defesa da educação e da saúde como direitos universais e bens públicos inalienáveis. Portanto, este texto (novas DCN/NF) é fruto do esforço coletivo, expressa o desejo de mudança na formação em enfermagem e traduz os avanços possíveis em um contexto de correlação de forças nem sempre favorável, mas que, ao mesmo tempo, impulsionou os movimentos e as lutas que resultaram nesta conquista histórica.

As novas DCN/ENF oportunizam refletir as profundas transformações sociais, sanitárias, científicas e educacionais da atualidade. Entre os principais avanços, destacam-se: a oferta do curso exclusivamente no formato presencial, conquista expressa no Decreto nº 12.456, de 19 de maio de 2025^(2)^, evidenciando a defesa histórica da ABEn pelo ensino presencial na enfermagem como um imperativo ético para a qualidade da formação; a afirmação do Sistema Único de Saúde como o eixo estruturante da formação; a assunção do cuidado de enfermagem como finalidade do trabalho da(o) enfermeira(o) e com foco nas necessidades de saúde da população, fortalecendo a identidade profissional da enfermagem; a incorporação de princípios que refletem as demandas contemporâneas por uma formação ética, crítica, política, socialmente referenciada e comprometida com a democracia, a cidadania, a dignidade humana e o enfrentamento das desigualdades e da exclusão sociais.

Ainda, as novas DCN/ENF definem cinco áreas de formação e conteúdos essenciais para a preservação da autonomia técnico-científica da(o) enfermeira(o), explicitando a necessidade de se garantir as bases filosóficas, teóricas e metodológicas na construção do Projeto Político-Pedagógico (PPP) dos cursos. Merece destaque também o reforço da indissociabilidade entre ensino, pesquisa e extensão, a integração ensino-serviço-comunidade e a articulação entre teoria e prática.

O rigor técnico-científico é enfatizado com vistas a promover uma formação de futuras(os) enfermeiras(os) capazes de responder às necessidades de saúde da população e às demandas dos territórios, reafirmando a enfermagem como prática social. Nesse sentido, as novas DCN/ENF apontam que o PPP deve promover o desenvolvimento de atividades de ensino-aprendizagem teóricas e atividades de ensino-aprendizagem teórico-práticas, destacando seu caráter integrado. É importante salientar que as atividades de ensino-aprendizagem teórico-práticas devem ocorrer desde o primeiro semestre do curso, em cenários reais e diversificados do mundo do trabalho, correspondendo a pelo menos 20% da carga horaria total do curso. O estágio supervisionado obrigatório, por sua vez, deve representar 30% da carga horária total do curso e ser realizado nos dois ou três últimos semestres.

No ano do centenário da ABEn, a publicação das novas DCN/ENF representa um avanço significativo e uma conquista histórica para a enfermagem brasileira, refletindo o compromisso e o protagonismo históricos da entidade nas questões relativas à educação e à formação em enfermagem. Essa conquista é resultado da mobilização e do esforço coletivo em prol da educação de qualidade, e também representa a resistência contra a mercantilização do ensino, reafirmando que não há vitória sem luta e que o futuro e a força da enfermagem dependem da mobilização contínua em defesa da vida, da saúde e da educação.

# Translation: Nuevas Directrices Curriculares para los Programas de Pregrado en Enfermería: un logro histórico para la enfermería brasileña

**DOI:** 10.1590/0034-7167.20267907es

**Authors:** Célia Alves Rozendo, Jacinta de Fatima Sena da Silva, Kênia Lara da Silva

**Affiliations:** I Universidade Federal de Alagoas. Directora Nacional de Educación de la Asociación Brasileña de Enfermería (Gestión 2025-2028). Alagoas, Maceió, Brasil; II Fiocruz Brasília, Docente de la Maestría en Políticas Públicas de Salud, Angicos y PSAT. Universidade de Brasília, Departamento de Salud Colectiva. Investigadora y Docente Colaboradora. Presidenta Nacional de la Asociación Brasileña de Enfermería (Gestión 2025-2028). Brasília, Distrito Federal, Brasil; III Universidade Federal de Minas Gerais, Escuela de Enfermería. Directora Nacional de Estudios e Investigación en Enfermería de la Asociación Brasileña de Enfermería (Gestión 2025-2028). Belo Horizonte, Minas Gerais, Brasil

En 2012, Belém do Pará fue el escenario del 13º Seminario Nacional de Directrices para la Educación en Enfermería (13º SENADEn - *Seminário Nacional de Diretrizes para a Educação em Enfermagem*), que marcó el inicio de las discusiones sobre la revisión de las Directrices Curriculares Nacionales para los Cursos de Pregrado en Enfermería (DCN/ENF- *Diretrizes Curriculares Nacionais para os Cursos de Graduação em Enfermagem*), cuyo tema central fue “Una Década de Directrices Curriculares Nacionales: logros y desafíos”. Transcurrió un largo período entre la realización de este SENADEn y la publicación de las nuevas DCN/ENF, ocurrida el 19 de mayo de 2026 mediante la Resolución CES/CNE nº 1/2026^(1)^.

Resultado de una amplia participación y de un debate colectivo bajo el liderazgo de la Asociación Brasileña de Enfermería (ABEn - *Associação Brasileira de Enfermagem*), este hito representa un logro fundamental para la enfermería, al establecer las bases para una formación de excelencia. Todo texto lleva consigo un contexto. Así, la construcción de las DCN/ENF, como todo proceso político, movilizó a diversos actores sociales, generó debates y enfrentó tensiones, disputas y desafíos en defensa de la educación y de la salud como derechos universales y bienes públicos inalienables. Por lo tanto, este texto (las nuevas DCN/ENF) es fruto del esfuerzo colectivo, expresa el deseo de transformar la formación en enfermería y refleja los avances posibles en un contexto de correlación de fuerzas no siempre favorable, pero que, al mismo tiempo, impulsó los movimientos y las luchas que culminaron en esta conquista histórica.

Las nuevas DCN/ENF permiten reflexionar sobre las profundas transformaciones sociales, sanitarias, científicas y educativas de la actualidad. Entre los principales avances se destacan: la oferta del curso exclusivamente en la modalidad presencial, logro consagrado en el Decreto nº 12.456, de 19 de mayo de 2025^(2)^, que evidencia la histórica defensa de la ABEn de la enseñanza presencial en enfermería como un imperativo ético para garantizar la calidad de la formación; la afirmación del Sistema Único de Salud como eje estructurante de la formación; la asunción del cuidado de enfermería como finalidad del trabajo de la enfermera y del enfermero, con énfasis en las necesidades de salud de la población, fortaleciendo la identidad profesional de la enfermería; y la incorporación de principios que responden a las demandas contemporáneas de una formación ética, crítica, política, socialmente comprometida y orientada a la defensa de la democracia, la ciudadanía, la dignidad humana y el enfrentamiento de las desigualdades y de la exclusión social.

Asimismo, las nuevas DCN/ENF definen cinco áreas de formación y contenidos esenciales para preservar la autonomía técnico-científica de la enfermera y del enfermero, explicitando la necesidad de garantizar las bases filosóficas, teóricas y metodológicas en la construcción del Proyecto Político-Pedagógico (PPP) de los cursos. También merece destacarse el fortalecimiento de la indisociabilidad entre la enseñanza, la investigación y la extensión, la integración enseñanza-servicio-comunidad y la articulación entre teoría y práctica.

Se enfatiza el rigor técnico-científico con el propósito de promover la formación de futuras enfermeras y futuros enfermeros capaces de responder a las necesidades de salud de la población y a las demandas de los territorios, reafirmando la enfermería como una práctica social. En este sentido, las nuevas DCN/ENF señalan que el PPP debe promover el desarrollo de actividades de enseñanza-aprendizaje teóricas y de actividades de enseñanza-aprendizaje teórico-prácticas, destacando su carácter integrado. Es importante señalar que las actividades de enseñanza-aprendizaje teórico-prácticas deben desarrollarse desde el primer semestre del curso, en escenarios reales y diversos del mundo laboral, y corresponder, como mínimo, al 20% de la carga horaria total del curso. Por su parte, la práctica supervisada obligatoria debe representar el 30% de la carga horaria total del curso y realizarse durante los dos o tres últimos semestres.

En el año del centenario de la ABEn, la publicación de las nuevas DCN/ENF representa un avance significativo y una conquista histórica para la enfermería brasileña, reflejando el compromiso y el protagonismo histórico de la entidad en las cuestiones relacionadas con la educación y la formación en enfermería. Este logro es resultado de la movilización y del esfuerzo colectivo en favor de una educación de calidad y también representa la resistencia frente a la mercantilización de la enseñanza, reafirmando que no hay victoria sin lucha y que el futuro y la fortaleza de la enfermería dependen de la movilización continua en defensa de la vida, la salud y la educación.
